# Comparative Transcriptome Analysis in Homo- and Hetero-Grafted Cucurbit Seedlings

**DOI:** 10.3389/fpls.2021.691069

**Published:** 2021-10-28

**Authors:** Filippos Bantis, George Tsiolas, Evangelia Mouchtaropoulou, Ioanna Tsompanoglou, Alexios N. Polidoros, Anagnostis Argiriou, Athanasios Koukounaras

**Affiliations:** ^1^School of Agriculture, Aristotle University of Thessaloniki, Thessaloniki, Greece; ^2^Centre for Research and Technology Hellas, Institute of Applied Biosciences, Thessaloniki, Greece; ^3^Department of Food Science and Nutrition, University of the Aegean, Myrina, Greece

**Keywords:** *Citrullus lanatus*, scion, rootstock, healing, RNA-seq

## Abstract

Watermelon (*Citrullus lanatus*) is a valuable horticultural crop with nutritional benefits grown worldwide. It is almost exclusively cultivated as grafted scions onto interspecific squash rootstock (*Cucurbita maxima* × *Cucurbita moschata*) to improve the growth and yield and to address the problems of soilborne diseases and abiotic stress factors. This study aimed to examine the effect of grafting (homo- and hetero-grafting) on the transcriptome level of the seedlings. Therefore, we compared homo-grafted watermelon (WW) with non-grafted watermelon control (W), homo-grafted squash (SS) with non-grafted squash control (S), hetero-grafted watermelon onto squash (WS) with SS, and WS with WW. Different numbers of differentially expressed genes (DEGs) were identified in each comparison. In total, 318 significant DEGs were detected between the transcriptomes of hetero-grafts and homo-grafts at 16 h after grafting. Overall, a significantly higher number of downregulated transcripts was detected among the DEGs. Only one gene showing increased expression related to the cytokinin synthesis was common in three out of four comparisons involving WS, SS, and S. The highest number of differentially expressed (DE) transcripts (433) was detected in the comparison between SS and S, followed by the 127 transcripts between WW and W. The study provides a description of the transcriptomic nature of homo- and hetero-grafted early responses, while the results provide a start point for the elucidation of the molecular mechanisms and candidate genes for the functional analyses of hetero-graft and homo-graft systems in *Cucurbitaceae* and generally in the plants.

## Introduction

Watermelon [*Citrullus lanatus* (Thunb.) Matsum. and Nakai] is a plant species cultivated for its highly nutritious fruits which contain about 92% water and 6% sugars, and are rich in vitamin C, lycopene, and citrulline (Erhirhie and Ekene, 2013). It is a valuable crop cultivated worldwide with over 100 million tons of annual production (Food and Agriculture Organization of the United Nations, 2012). Nevertheless, watermelon cultivation can be limited because of its sensitivity to soil-borne diseases, low and high temperatures, organic pollutants, heavy metals, high salinity, drought, and insect pests (Garcia-Lozano et al., 2020). In Western European countries and Greece, vegetable grafting has become of interest in the last decade, especially due to the ban of methyl bromide as a soil disinfectant in 2005 and the high demand for environmentally friendly products (Davis et al., 2008). Grafting is the main propagation technique in many leading countries for watermelon production, such as South Korea, Japan, Spain, Italy, and Greece. In 2005, over 90% of the grafted watermelon seedlings were used in Japan and Korea and the production exceeded 300 million grafted seedlings. In 2009, Spain and Italy jointly produced 60 million grafted watermelon seedlings (Lee et al., 2010), while in Greece about 19 million grafted watermelon seedlings are produced annually (Bantis et al., 2019). High compatibility can be achieved between the watermelon scions and watermelon rootstocks (Fallik et al., 2019) or interspecific squash hybrid rootstocks, such as TZ-148 (*Cucurbita moschata* × *C. maxima*) which is the most common rootstock for the grafted watermelon seedlings throughout the world (Lee et al., 2010).

Vegetable grafting is a propagation technique that offers several benefits during crop production. The technique involves two segments, the scion and the rootstock, which conjoin and form a grafted seedling with desired features. Briefly, grafting increases the plant resistance against biotic (i.e., soil-borne pathogens) (Lee et al., 2010; Louws et al., 2010) and abiotic (i.e., heavy metals, salinity, and low temperatures) (Savvas et al., 2010; Schwarz et al., 2010) stress factors, improves the fruit quality and ripening behavior (Soteriou et al., 2014), and enhances plant vigor due to the higher rootstock activity (Savvas et al., 2010). Grafting is characterized as “homo-grafting” when the scion and rootstock are seedlings from the same species, or as “hetero-grafting” when the scion and rootstock are seedlings from different species (Yeoman and Brown, 1976). Biological molecules, such as nutrients, hormones, proteins, and genetic material are transported through graft fusion due to the interaction between the two graft segments. Several types of research have been conducted, revealing the molecular responses at the grafting site and there is significant evidence that the movement of genetic material (organelle and nuclear DNA) at the grafting site and the grafting induced changes are stably inherited in the subsequent generations (Taller et al., 1999; Stegemann and Bock, 2009; Stegemann et al., 2012; Tsaballa et al., 2013; Fuentes et al., 2014). Stegemann and Bock (2009) were the first who proposed that grafting provides a path for horizontal gene transfer (HGT), based on their experimental studies in which after grafting of genetically modified tobacco plants, plastid genes were able to travel over the grafting point, noting that the gene transfer is confined to the graft site and no long-distance transfer may occur and “graft hybridization” would not be analogous to the sexual hybridization. On the contrary, Fuentes et al. (2014) detected nuclear genome transfer between the scion and rootstock resulting in new fertile and stable allopolyploid species, thus proposing that grafting should be used as an alternative way of polyploidization, while interspecies grafting could allow a method to produce new allopolyploid crop species, offering a remarkable potential in breeding (Fuentes et al., 2014). Beyond the DNA movement across the graft union, there is numerous research associated with epigenetic modification in the grafted plants regulated by the transmittable small RNA, miRNA, mRNA, and proteins (Sharma and Zheng, 2019).

The process of successful grafting depends on wound healing, following this order: wound response, cell regeneration, cell proliferation, cell-cell adhesion, and cell differentiation (Goldschmidt, 2014; Gaut et al., 2019). The plants use the vascular tissue (xylem and phloem) to transport water, nutrients, photosynthetic products, and signaling molecules, such as plant hormones, and to provide mechanical support (Nanda and Melnyk, 2018). The vascular adhesion process has been well-studied, but the molecular mechanisms underlying this process remain not fully understood. It has been established, however, that the plant hormones, such as auxins, cytokinins (CKs), ethylene (ET), gibberellins (GAs), and jasmonic acid (JA) play a crucial role in the regulation of physiological processes taking place at the graft junction (Melnyk et al., 2015; Ikeuchi et al., 2017; Nanda and Melnyk, 2018). Specifically, the plant hormones are the signaling molecules, responding to either artificial or natural plant wounds. Regarding the wound response promoted by CK and by the WOUND INDUCED DEDIFFERENTIATION 1 (WIND1) pathway, which is upregulated upon wounding, and the overexpression of this gene results in excess callus formation (Iwase et al., 2011). A plethora of research has proved the vital role of auxin in the process of graft union (Sharma and Zheng, 2019), and the results from the work of Melnyk et al. (2015) enhance the significance of auxin in vascular formation, such as a new role for ALF4.

To determine the gene expression and related transcriptional networks and the metabolic activities pertinent to successful grafting, we examined the transcription in different grafting combinations between the interspecific squash rootstock and watermelon scion. In this study, we used controls of the non-grafted seedlings and homo-grafted squash and watermelon to examine the alteration of gene expression in watermelon-squash hetero-grafts. The transcriptomic data were analyzed using bioinformatic tools to detect the differentially expressed genes (DEGs) during the early stages of the healing process. A significant hormone regulating gene (LOG5) detected in the grafting combinations involving squash as rootstock was further validated using a quantitative real-time PCR (qPCR) analysis. This study provides a description of potential groundwork for the elucidation of critical steps in the healing process of the commercially important watermelon scion and interspecific squash rootstock grafting combinations.

## Materials and Methods

### Plant Material and Growth Conditions

The experiment was conducted in Kleidi, Imathia, Greece, in the facilities of Agris S.A.^1^ A transcriptome analysis was performed at the Institute of Applied Biosciences of the Centre for Research and Technology Hellas, Thessaloniki, Greece.

The grafted seedlings were composed of watermelon [*C. lanatus* (Thumb.) Matsum. and Nakai] “Celine F1” (regularly used as scions) and interspecific squash (*Cucurbita maxima* × *Cucurbita moschata*, hereafter referred to as squash) “TZ-148” (regularly used as rootstocks) combinations. The watermelon and squash seeds (HM.Clause SA, Portes-Les-Valence, France) were sown in 171-cell plug trays and 128-cell plug trays (67 cm × 33 cm, G.K. Rizakos S.A., Lamia, Greece), respectively. The trays were filled with peat, perlite, and vermiculite (5:1:2).

Upon sowing, the trays remained in a chamber with controlled conditions (temperature 25°C, relative humidity 95–98%, and darkness) for 72 h (watermelon) or 48 h (squash) to assist germination. After germination, the watermelon trays were moved for 10 days in a greenhouse with 21.5°C minimum night temperature and supplemental artificial lighting emitted by the high-pressure sodium lamps (MASTER GreenPower 600 W 400 V E40, Philips Lighting, Eindhoven, The Netherlands) at photosynthetic photon flux density (PPFD) of 100 ± 10 μmol m^–2^ s^–1^ and 18 h photoperiod. In parallel, the squash trays were moved in a greenhouse under 20°C minimum night temperature for 7 days, and afterward 14°C for 3 days to reduce the growth rate and increase the stem thickness. According to the previous experiments of our group, no supplemental artificial lighting was needed for the production of squash seedlings.

### Grafting Combinations

The “splice grafting” technique was employed when the watermelon and squash had developed one true leaf. Table 1 depicts the plant material used in this study. Briefly, the material included non-grafted controls: watermelon (W) and squash (S), homo-grafted watermelon scion grafted onto watermelon rootstock (WW), and squash scion grafted onto squash rootstock (SS); and the hetero-grafted watermelon scion grafted onto squash rootstock (WS). The segments of the grafted seedlings were held together with a silicon clip and planted in 72–cell plug trays (50 cm × 30 cm) filled with peat, perlite, and vermiculite (3:1:1). Grafting was performed by an experienced person for the avoidance of critical errors. Immediately after grafting, the seedlings from all the combinations were moved in a healing chamber under 25°C temperature, 98% relative humidity, recirculating air, and sole artificial lighting emitted by the fluorescent lamps (Fluora 58 W, Osram, GmbH, Munich, Germany) at PPFD of 45 ± 5 μmol m^–2^ s^–1^ and 16 h photoperiod.

**TABLE 1:** Watermelon and interspecific squash grafting combinations tested after 16 h in a healing chamber.

Abbreviation	Scion		Rootstock	Grafting combination
W	Watermelon		–	Non-grafted
S	Int. squash		–	Non-grafted
WS	Watermelon	×	Int. squash	Hetero-grafted
WW	Watermelon	×	Watermelon	Homo-grafted
SS	Int. squash	×	Int. squash	Homo-grafted

### Sampling

The sampling was conducted after 8 h of light and 8 h of darkness (in total 16 h) in the healing chamber in almost complete darkness. Specifically, the segments from 15 seedlings per combination were cross-cut with the sterilized blades 0.5 cm above and below the grafting junction. The segments from the non-grafted seedlings were cut precisely below the cotyledons. After cutting, each segment from all the combinations was immediately wrapped with aluminum foil, submersed into liquid nitrogen, and placed in deep freezing temperature (−80°C) until molecular analysis.

In the previous (unpublished) experiments of our group, such as microscopy and phytohormonal analysis on the third day after grafting, we concluded that vascular reconnection starts earlier than the third day after grafting. Since phytohormonal activity begins earlier than the third day, molecular activity, such as gene expression and signaling initiates earlier. Moreover, in the first few hours after grafting, the wounding effect takes place, thus we opted to avoid the sampling until 8 h after grafting. Therefore, we selected the 16-h time point which lies between 8 h and 3 days after grafting to avoid the wounding effect and not miss the gene expression during the first crucial day.

### RNA Isolation, Library Preparation, and Sequencing

Total RNA was extracted from the grafting junctions with NucleoSpin RNA Plant, Mini kit for RNA from the plant (Macherey-Nagel GmbH & Co. KG, Germany) from the three biological replicates of each treatment according to the instructions from the manufacturer. The total yield was quantified by a fluorometric method with Qubit^TM^ RNA BR Assay kit (Cat. No. Q10211, Thermo Fischer Scientific, MA, United States), and the integrity of the isolated genetic material was accessed with agarose gel electrophoresis. For the library construction, the mRNA was purified with oligo-dT^(25)^ magnetic beads (Cat. No. S1419S, New England Biolabs, MA, United States). The libraries were prepared with the NEBNext^®^ Ultra^TM^ II RNA Library Prep Kit for Illumina^®^ (Cat. No. E7770S, New England Biolabs) according to the instructions from the manufacturer, with an average insert size of 300 base pair (bp). The size of libraries was estimated by capillary electrophoresis with the 5400 Fragment Analyzer system (Agilent, CA, United States) and the final quantification performed by qPCR using the KAPA Library Quantification kit for Illumina^®^ sequencing platforms (Cat. No. KK4824, Roche, Switzerland) on a Rotor−Gene Q thermocycler (Qiagen, Germany). The libraries were sequenced with Illumina^®^ Nexteq500^®^ platform using the NextSeq^®^ 500/550 v2.5 High Output Kit (300 cycles) (Cat. No. 20024908, Illumina, CA, United States).

### Bioinformatic Analysis

**FIGURE 1:**
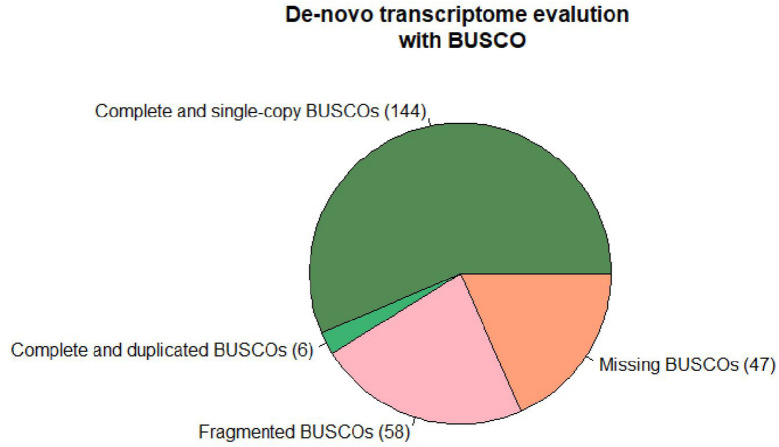
Results of transcriptome assembly completeness calculated with BUSCO.

**FIGURE 2:**
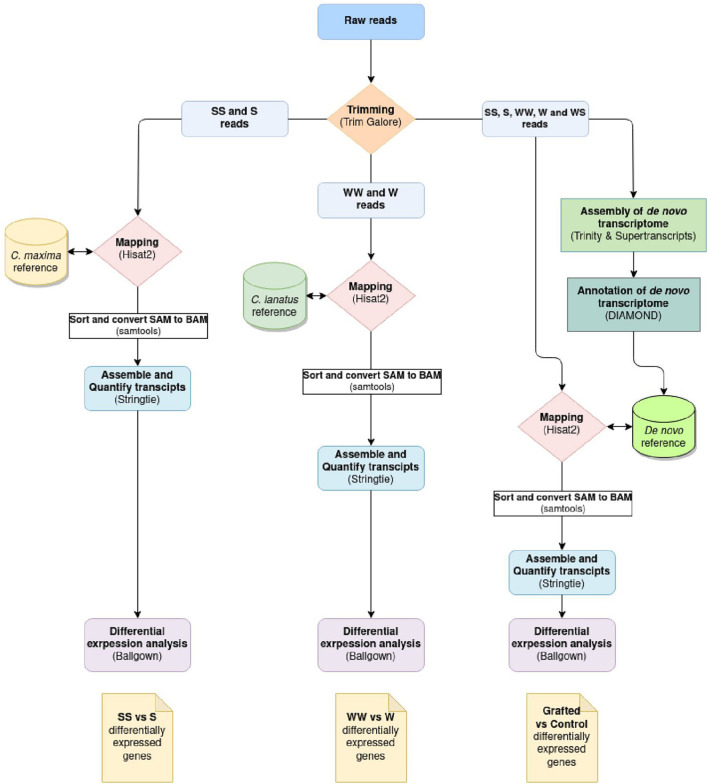
Differential expression analysis workflow. The same workflow was followed for all the three comparisons, which differ only at the reference used for the mapping of the reads.

The differential expression analysis was performed according to the “New Tuxedo” pipeline (Kim et al., 2016). The raw reads filtered with Trim Galore!^2^ for quality (q 28), length (min-length 100), and the sequencing adaptors. The filtered reads of the samples SS and S were aligned to the *C. maxima* genome (GCA_002738345.1) and then of the samples WW and W to the *C. lanatus* genome (GCA_004801215.2) using Hisat2.^3^ Stringtie^4^ was used for the assembly and quantification of transcripts. Differential expression analysis was performed with Ballgown^5^ in R studio. In parallel, a *de novo* transcriptome of the total reads acquired from the different samples was assembled with Trinity^6^ to be used as a reference for the mapping of the reads of different organisms. The assembly completeness was evaluated with BUSCO^7^ (Figure 1). A genome-like reference file was created with Supetranscripts, a module of Trinity assembler, which was used for the downstream analysis following the same protocol described above. The *de novo* transcriptome was annotated with Diamond.^8^ The analysis pipeline is depicted in Figure 2. The raw Illumina reads are available under the Bioproject PRJNA721571.^9^

### Quantitative Real-Time PCR Analysis

The total RNA from the segments of homo-grafted WW, SS, hetero-grafted WS, and non-grafted W and S, which have been harvested at 0 (control), 16, and 24 h after grafting in the growth chamber with the three biological replicates was extracted using a total RNA isolation kit (Monarch^®^ Total RNA Miniprep Kit, NEB) following the instructions from the manufacturer. All the samples were treated by DNase I for enzymatic removal of residual gDNA. To determine the RNA quality and concentration, 5 μl of each RNA sample was analyzed by agarose gel electrophoresis (1.4%, agarose, 1 × TBE) and quantified using a NanoPhotometer IMPLEN Version 7122 V2.3. The reverse transcription and PCR reactions were performed using a Luna^®^ Universal One-Step reverse transcription-quantitative PCR (RT-qPCR) Kit (E3005) following the instructions from the manufacturer with the following steps: total volume for each reaction was 20 μl, including 10 μl Luna Universal One-Step Reaction Mix (2×), 1 μl Luna WarmStart^®^ RT Enzyme Mix (20×), 0.8 μl forward and reverse primers of LOG5, while 0.2 μl forward and reverse primers for actin, 20 ng of RNA and nuclease-free water up to 20 μl. The Roche LightCycler^®^ 96 System was used with the following thermal cycling conditions: stage 1 for RT, 1 cycle at 55°C for 10 min; stage 2, 1 cycle at 95°C for 1 min; stage 3, 40 cycles at 95°C for 10 s and 60°C for 30 s. After amplification, a melting curve analysis was performed to verify the product. At least three biological replicates were performed. The measured Ct values were converted to relative copy numbers using the ΔΔCt method. The primer pairs were for Log5 forward 5′CATCCACGACAAACCAGTTG3′ and reverse 5′AGGCACGTACTCCTCTAGTTTCTG3′ amplifying a 172 bp product and for actin forward 5′CCATGTATGTTGCCATCCAG3′ and reverse 5′GGATAGCATGGGGTAGAGCA3′ amplifying a 140 bp product. The primers for the reference gene CIACT (gene ID Cla007792) were based on the published data (Kong et al., 2014), and the primers for LOG5 (gene ID LOC111776908) were designed to amplify a specific sequence region common in the watermelon and squash (based on the published consensus sequences in the Cucurbit Genome Database).^10^ The results were analyzed using the Roche LightCycler^®^ 96 software program, version 1.1.

## Results

### RNA-Seq Data Analysis

In total 510 million sequence reads were obtained, corresponding to approximately 17 million raw reads for each sample. After quality filtering, about 42 million of them were excluded from the following analysis due to low quality. The filtered reads of the samples SS and S were aligned to the *C. maxima* genome and those of the samples WW and W to the *C. lanatus* genome as described in the section “Materials and Methods.” The overall alignment rate was ranging from 69 to 97% and the total number of genes identified was 29,374 for the grafted squash samples and 21,775 for the watermelon samples (Table 2).

**TABLE 2:** Discovered genes and transcripts with “New Tuxedo” protocols of the three comparisons.

	*C. maxima* reference	*C. lanatus* reference	*De novo* transcriptome
Total genes	29,374	21,775	192,133
Total transcripts	59,924	41,119	–
DE genes	238	31	318
DE transcripts	433	127	–

### Differentially Expressed Genes

**FIGURE 3:**
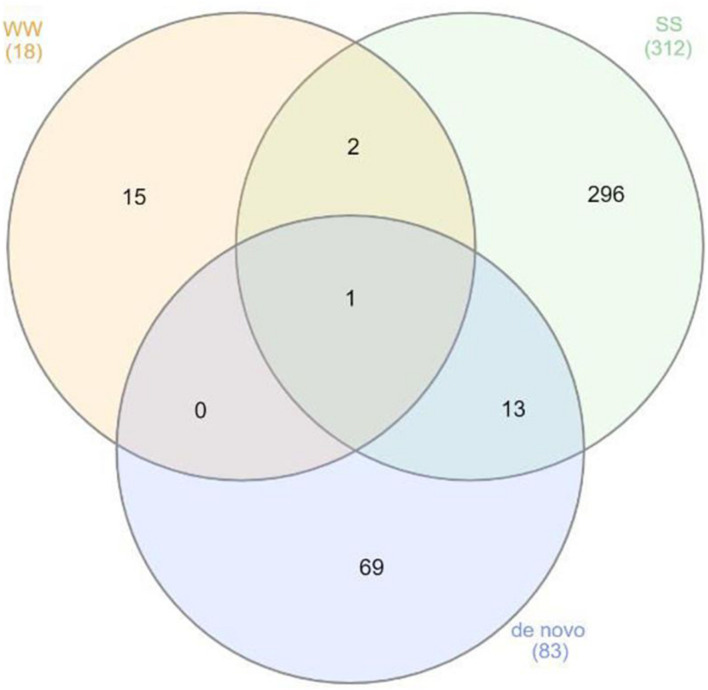
Venn diagram depicting the gene ontologies found to be differentially expressed (DE) among the three comparisons.

**FIGURE 4:**
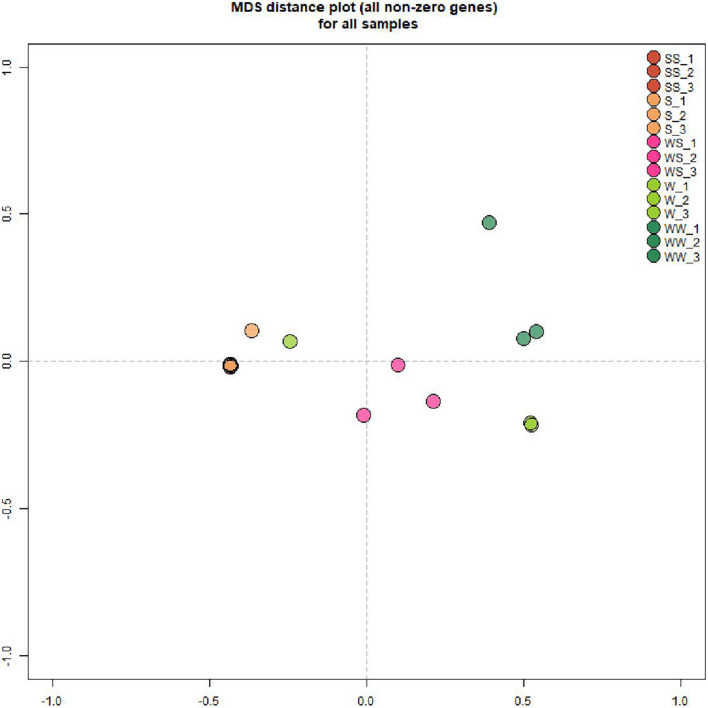
Multidimensional Scaling (MDS) plots for grafted plants (WW, SS, an WS) vs. non-grafted controls (W, S). Worth mentioning are the results of WS samples which are positioned between squash and watermelon samples.

**FIGURE 5:**
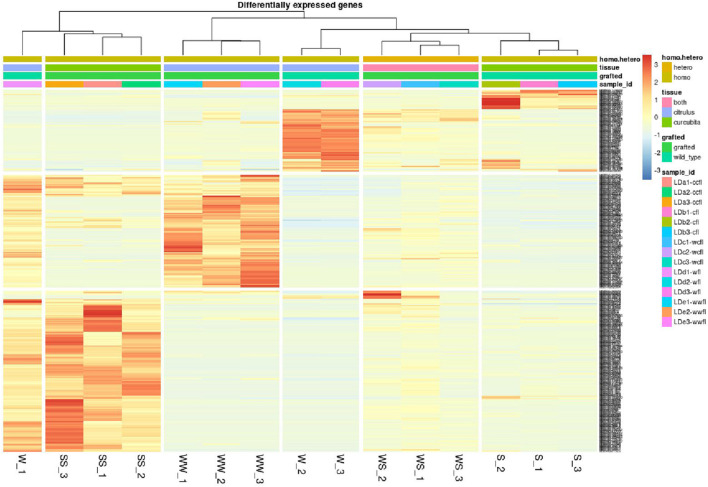
Heatmap of differentially expressed genes (DEGs) of grafted plants (WW, SS, an WS) vs. non-grafted controls (W, S). The heatmaps depict genes with *p* < 0.05 and fold change > 4. The color scale (blue to red) represents the expression levels in FPKM.

To assess the effect of grafting in homo-grafted squash and watermelon, differential expression analysis was performed. Among the homo-grafted squash (SS), the biological replicates and the non-grafted plants (S) that were used as controls (comparison SS/S), 238 genes, and 433 transcripts were found to be differentially expressed (DE). Similarly, among the homo-grafted watermelon replicates (WW) and their control (W) replicates (comparison WW/W), 31 genes and 127 transcripts were found to be DE. The comparison among the grafted plants, homo- and hetero-grafted, and their controls (comparison WS + SS + WW/W + S) revealed 318 DEGs (Table 2). A Venn diagram depicting the number of DEGs among the three comparisons is shown in Figure 3. The detection of DE transcripts was not feasible since, due to the adopted methodology, a *de novo* transcriptome, was built and used as a reference. This transcriptome consists of transcripts that are highly conserved among the two species and unique transcripts that are expressed only in one of the two species. Although the *de novo* transcriptome method offers a way to investigate the genes that were affected at the hetero-grafted plants during the process of healing, we cannot be confident about their levels of expression. The two additional comparisons were performed for the hetero-grafted plants (WS) against the two homo-grafted (WW and SS). The comparison WS/WW identified 36 and the comparison WS/SS identified 214 DE transcripts. The multidimensional scaling (MDS) plots for grafted plants (WW, SS, and WS) vs. non-grafted controls (W and S) in Figure 4 and a heatmap of DEGs in Figure 5 support this finding. The expression levels of WS samples do not show the fluctuation that is found in WW and SS samples, which is caused by the bioinformatic analysis method where the *de novo* transcriptome was used as reference.

### Functional Analysis of Differentially Expressed Genes

The assessment of the possible functions of the DEGs was feasible only for the homo-graft comparisons. The analysis revealed that the most common DEGs among WW, SS, and their controls are related to the specific biological processes, mainly associated with the stress responses (Table 3). Similarly, DEGs were found to be commonly over- and under-expressed among the three comparisons of homo- and hetero-grafted plants vs. their control seedlings. Again, the most commonly overexpressed genes (Table 4) are related to known biological mechanisms involved in the stress response.

**TABLE 3:** The gene ontologies of mutually differentially expressed genes (DEGs) of homo-grafted watermelon (WW) and homo-grafted squash (SS).

Gene ontologies of DEGs					
Biological Process		Molecular Function		Cellular Component	
GO:0006950	Response to stress	GO:0016787	Motor activity	GO:0005576	Extracellular region
GO:0010466	Negative regulation of peptidase activity	GO:0016798	Hydrolase activity, acting on glycosyl bonds	GO:0048046	Apoplast
GO:0010951	Negative regulation of Endopeptidase activity	GO:0016491	Oxidoreductase activity	GO:0016020	Membrane
GO:0005975	Carbohydrate metabolic process	GO:0050660	Flavin adenine dinucleotide binding	GO:0016021	Integral component of membrane
GO:0008152	Metabolic process	GO:0015238	Xenobiotic transmembrane transporter activity	GO:0009507	Chloroplast
GO:0071555	Cell wall organization	GO:0015297	Antiporter activity	GO:0005886	Plasma membrane
GO:0055114	Obsolete oxidation-reduction process	GO:0004601	Peroxidase activity		
GO:0006855	Drug transmembrane transport	GO:0020037	Heme binding		
GO:0055085	Transmembrane transport	GO:0046872	Metal ion binding		
GO:0006979	Response to oxidative stress	GO:0005509	Calcium ion binding		
GO:0042744	Hydrogen peroxide catabolic process	GO:0003824	Catalytic activity		
GO:0006355	Regulation of transcription, DNA-templated	GO:0005524	ATP binding		
GO:0008643	Carbohydrate transport	GO:0008289	Lipid binding		
GO:0006090	Pyruvate metabolic process	GO:0016740	Transfer activity		
GO:0016310	Phosphorylation				
GO:0006952	Defense response				
GO:0009607	Response to biotic stimulus				
GO:0006869	Lipid transport				

**TABLE 4:** Genes were found to be differentially expressed (DE) among the comparisons. No common DEGs were found among the comparisons of WW, hetero-grafted watermelon onto squash (WS), and their controls.

WW and SS vs. controls	SS and WS vs. controls	WW, SS, and WS vs. controls
**Over-expressed**		
Bidirectional sugar transporter SWEET	Cinnamyl alcohol dehydrogenase 1-like	Dehydrin
Gibberellin-regulated family protein	Ethylene-responsive transcription factor	Disease resistance-responsive (Dirigent-like protein) family protein
Lipid transfer protein	GDSL esterase/lipase	Peroxidase
Pathogenesis-related protein 1	Glutamate decarboxylase	
	Lactoylglutathione lyase	
	Non-specific lipid-transfer protein	
	Pectate lyase	
	Pectinesterase	
	Sugar transporter, putative	
	Xyloglucan endotransglucosylase/hydrolase	
**Under-expressed**		
	BURP domain-containing protein	
	Stem-specific protein TSJT1-like	

### Validation of LOG5 Expression by RT-qPCR Analysis

**FIGURE 6:**
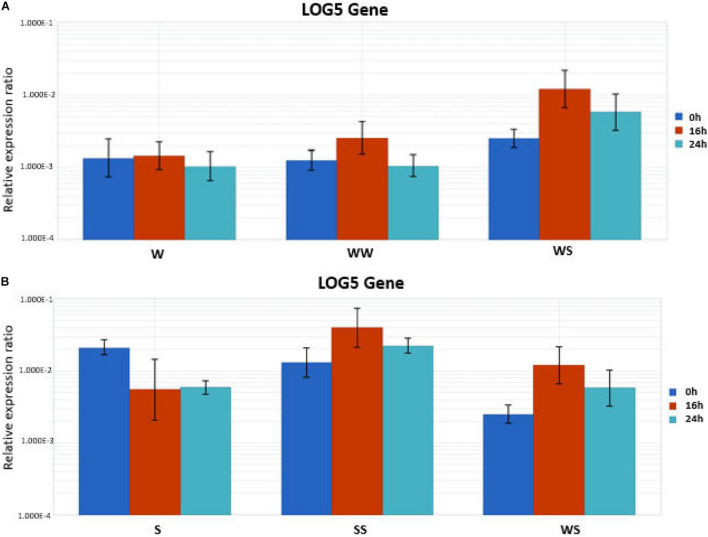
Quantitative real time RT-PCR gene expression analysis of LOG5 in stems (including the grafting junction). **(A)** W (watermelon seedling stem), WW (homo-grafted watermelon), and WS (hetero-grafted watermelon scion on squash rootstock) and **(B)** S (squash seedling stem), SS (homo-grafted watermelon), and WS (hetero-grafted watermelon scion on squash rootstock) at three different time points after grafting.

To validate the RNA-Seq expression data for LOG5 that showed relatively high abundance in three out of the four comparisons, we performed RT-qPCR analysis. Total RNA from the stem of seedlings and the graft region including tissue from the rootstock and the scion of homo- and hetero-graft combinations was used as the templates. The RT-qPCR results were consistent with an upregulation of LOG5 found in the RNA-seq analysis. Relative LOG5 expression was stable in the stem of watermelon seedlings after transfer to the growth chamber while showed a decrease in squash (Figure 6). An increase of LOG5 transcript level was detected at 16 h in all the grafting combinations, which was higher in homo-grafted SS followed by the hetero-grafted WS and homo-grafted WW. Later, at 24 h, the increase was at a lower level in WS and SS while in WW LOG5, the expression was similar to the non-grafted control.

## Discussion

Watermelon is considered an economically important crop, especially in eastern Asia and the Mediterranean region. As soil-borne pathogens are virtually impossible to control, grafting has already provided an efficient, among the other benefits, solution for the successful watermelon establishment and cultivation in the field (Kyriacou and Soteriou, 2015). The unpublished observations of our group clearly showed that the process of healing for the grafted watermelon seedlings is characterized by rapid vascular reconnection only 3 days after grafting which is significantly faster than tomato (c.f. Wang et al., 2019) in which the scion and rootstock reconnection was observed after 9 days (Cui et al., 2021). Upon its formation, callus rapidly differentiates in the vascular tissues (i.e., phloem and xylem), while plasmodesmata, the intracellular channels, form between the scion and rootstock cells.

During the healing and tissue reconnection in grafted plants, there are critical molecular events characterized by differential gene expression. These events include the activation of wounding stress responses, such as biosynthesis of the stress-related phytohormones jasmonic acid and ethylene (Yin et al., 2012), and increased expression of ROS scavenging enzymes. The early responses also elicit auxin and brassinosteroid biosynthesis and sugar transport above the cut, increased stress response gene expression below the cut (Xie et al., 2019), and the activation of auxin and CK responses in the vascular cambium and pericycle (Melnyk et al., 2015). Accordingly, our data show differential regulation of genes involved in the wound and stress responses, hormonal signaling, and metabolite transport.

The stress responses are among the major biological processes identified by the DEGs among the homo-grafted WW and SS plants and their respective non-grafted controls. Mutually DEGs of WW and SS are involved in the response to stress (GO:0006950), response to oxidative stress (GO: 0006979), hydrogen peroxide catabolic process (GO:0042744), with molecular functions, such as oxidoreductase activity (GO:0016491) and peroxidase activity (GO:0004601). Similarly, in tomato genes involved in response to the oxidative stress (GO: 0006979), hydrogen peroxide catabolic process (GO: 0042744), and cellular oxidative detoxification (GO: 0098869) were enriched in the grafted plants (Xie et al., 2019). Additionally, in tomatoes, the oxidative detoxification enzymes accumulate to high levels near the graft region (Fernandez-Garcia et al., 2004) indicating that the differential gene expression is linked to function. The plant peroxidases are involved in the important developmental stages, such as lignification, auxin metabolism, reactive oxygen species (ROS) metabolism, and cell wall metabolism (Pandey et al., 2017). Fernandez-Garcia et al. (2004) reported that the peroxidases might be involved in the development of graft union in tomato plants. Similarly, in our study, the peroxidases were found to be over-expressed in all the graft combinations (WW, SS, and WS) compared with the non-grafted watermelon seedlings. Ascorbic acid (AsA) is the most abundant water-soluble antioxidant in the plants, with a significant role in the detoxification of ROS and regulating the cellular redox potential (Mittler, 2002; Suza et al., 2010; Fotopoulos and Kanellis, 2013). AsA is a critical cofactor of many dioxygenases that are related to the key steps in cell metabolism, affecting cell division and expansion (Fiorani et al., 2013). Therefore, the plants need AsA during the healing process after grafting. Wadano et al. (1999) reported that AsA increased sharply up to 8 h after grafting compared with control (non-grafted seedlings), while 30 h after grafting the content of the compound decreased to the level of control. Smirnoff (1996) showed that the AsA is required in the plants recovering from wounding. The plant signaling during grafting and wounding may share similar mechanisms, thus, a reasonable assumption is that the signal to increase AsA content may originate from the cell wall metabolism and cell expansion. In our study, two genes related to AsA transport, a nucleobase-AsA transporter 12-like, and an AsA transporter, were DE between the WS and SS grafted seedlings. AsA transporter is reported to facilitate the AsA transport from the cytosol to the chloroplast (Miyaji et al., 2015), a necessary activity for the AsA intracellular distribution (Maurino et al., 2006).

The plant signal transduction pathways are interconnected and form a network that is finely regulated by the plant hormones (Liu et al., 2016). The auxins, gibberellins, CKs, abscisic acid, ethylene, and other plant hormones participate in the plant stress defense mechanisms (Han and Kahmann, 2019). In our study, several differentially regulated hormone-related genes were identified. These responses signify the role of different hormone pathways, such as auxin, gibberellic acid, ethylene, and CKs in the process of graft vascular connection. An auxin-regulated gene expression modulates plant growth and development. Similarly, the CK signaling promotes callus formation by manipulating the cell cycle proteins (Ikeuchi et al., 2017). The auxin and CK response are strongly enhanced in the pericycle and vascular cambium of the grafted Arabidopsis plants (Melnyk et al., 2015). The transcripts related to auxin transport (auxin efflux carrier component 5) and signaling (auxin-binding protein ABP19a-like) were upregulated in the SS/S comparison while an auxin transporter-like protein 1 was upregulated in WS compared with the WW grafted plants. The CKs regulate the vascular tissue adhesion-dependent upon the working of signaling receptors, such as AHK2, AHK3, and CKI1 His kinase (CYTOKININ INDEPENDENT 1) (Sharma and Zheng, 2019). Our results showed upregulation of the CK biosynthetic gene LOG5 possibly triggered in the plants during the process of wound healing, proposing the involvement of CK signaling in the graft development. The CKs are known as key regulators of plant growth and development, controlling proceedings, such as cell division, growth of shoot apical meristem, development of the vascular system, root growth, tissue patterning, and shoot organogenesis (Sharma and Zheng, 2019). Historically, the CKs are regarded as root synthesized (Ghanem et al., 2011), although it has been revealed that the direct activation pathway *via* LOGs genes plays a pivotal role in regulating the CK activity during normal growth and development in Arabidopsis and rice (Kurakawa et al., 2007; Kuroha et al., 2009). Accordingly, our results demonstrated an increase of LOG5 transcript level in all the grafting combinations, which was higher in the homo-grafted SS followed by the hetero-grafted WS and homo-grafted WW.

A gibberellin-regulated protein (GRP) gene was overexpressed in the WW and SS grafted seedlings compared with the control treatments. The expression of this gene is shown to be upregulated by the gibberellins which are important for plant growth and development (Muhammad et al., 2019), but the function of the GRP is not clear.

Ethylene is an important hormone that participates in various activities related to plant growth and development (Ju and Chang, 2015). In our study, the ethylene-responsive transcription factors (ERFs) ERF115, ERF114-like, ERF113-like, and ERF110-like were over-expressed in WS and SS where the root system was removed, but not in WW where the root system was intact. The ERF family proteins are known for their ability to induce abiotic stress resistance in plants (Debbarma et al., 2019). Specifically, an ERF115 is demonstrated as a repressor of adventitious root initiation through the activation of CK and jasmonic acid signaling, the latter in NINJA-dependent and independent manners (Lakehal et al., 2020). An ERF114 is involved in the control of axillary bud outgrowth and cell proliferation, and activates the genes of the cell cycle and dormancy breaking, while it downregulates genes related to the cell wall-remodeling (Mehrnia et al., 2013). An ERF113 is a transcriptional activator participating in tolerance to the abiotic stress factors and plant development (Krishnaswamy et al., 2011). An ERF110 is assumed to act as a transcriptional activator through binding to the GCC-box pathogenesis-related promoter and is considered to participate in the stress-related gene expression patterns (Tao et al., 2018). Moreover, APETALA2/ERF (AP2/ERF) and xyloglucan endotransglucosylase/hydrolase were also upregulated in WS and SS compared with the non-grafted seedlings. AP2/ERF is the second largest family of TFs in the plants (Nakano et al., 2006). Quite similar to our findings, Spano et al. (2020) reported upregulated AP2/ERF and xyloglucan hydrolase in the self-grafted tomato plants. The abovementioned results of the transcriptional analysis of the grafted watermelon seedlings might provide an understanding of the interactions between the ERF genes and ethylene-dependent mechanisms during the healing process of watermelon grafted on interspecific squash.

Two genes, stem-specific TSJT1-like and BURB domain-containing protein were under-expressed in SS and WS compared with the non-grafted seedlings. TSJT1 is suggested to act as a negative regulator of internode development in the castor plants, but the specific function of protein is not clear (Hu et al., 2016). Quite similarly with our findings, Zhang et al. (2018) reported that a stem-specific protein TSJT1-like (*IpSR9*) showed decreased expression patterns under salt and osmotic stress. The BURP domain-containing proteins impose variable effects related to the plant development and responses to abiotic stress factors, but knowledge about this protein family is rather scarce (Wang et al., 2015). However, it is known that they are responsive to the application of abscisic acid (Matus et al., 2014). Gautier et al. (2020) found that a BURP domain-containing protein was DE as a response to grapevine hetero-grafting in the two different rootstocks.

Recently, the SWEETs (Sugars Will Eventually be Exported Transporters) are identified as the cellular sugar transporters which enable sucrose efflux through the cell membranes, from the phloem parenchyma to the phloem apoplasm (Chen, 2014). Phloem and xylem, the two major vascular tissue types, are probably the most important tissues that quickly differentiate during the healing of grafted seedlings (Melnyk and Meyerowitz, 2015). Only recently, a total of 17 CsSWEET genes were identified in another cucurbit, cucumber (Hu et al., 2017). The same authors reported that most of the CsSWEET genes were related to tissue development, while 18 ClaSWEET genes from watermelon and 18 CmSWEET genes from melon were similar to the CsSWEET genes from cucumber. In our study, a SWEET16-like gene was overexpressed in WW and SS, compared with the non-grafted seedlings. SWEET16 is found to facilitate sugar efflux from the vacuoles while its regulation is critical for development under unfavorable conditions (Klemens et al., 2013). These results provide additional information about the regulation and activity of SWEET genes in response to grafting, a very stressful procedure for the plants.

In summary, our results suggest that at the 16 h time-point, the majority of the DEGs are implicated in wound healing, stress response, hormone biosynthesis, transport and signaling, and metabolite transport. The common genes that were found to be DE among the hetero- and homo-grafted seedlings suggest the presence of “core genes” that are responsible for healing in the grafting procedure *per se*, species independently. Furthermore, the common DEGs found among SS and WS give an insight into the genes that are probably regulated only in the rootstock. These findings broaden our understanding of the molecular events that pertain to the successful grafting in *Cucurbitaceae* and deliver new questions that necessitate further investigation—including a new experimental approach—to obtain a distinct profile of genes that regulate the connection of scion and rootstock in grafting.

## Conclusion

In the present study, we performed a comprehensive transcriptomic analysis to examine the effect of grafting in the watermelon-squash scion-rootstock combinations. The control comparisons were performed to examine the effects of homo-grafting. The comparison between SS and S exhibited the highest number of DE transcripts, while WW and W comparison followed. Squash had a more abrupt response to grafting compared with watermelon as shown by the differential gene expression. To examine the effects of grafting in the hetero-grafted seedlings a *de novo* transcriptome, such as the genes from watermelon and squash, was assembled and used as a reference. Although the *de novo* transcriptome method offers a way to investigate the genes that were affected at the hetero-grafted plants during the process of healing, the results should be used with caution regarding the levels of expression. Among the overexpressed genes, only one gene (LOG5) was common in three out of four comparisons involving WS, SS, and S and is related to the CK synthesis. Its expression levels were precisely quantified and the results concur with the transcriptomic data. The study is a description of the transcriptomic nature of homo- and hetero-grafted early responses, while it provides a starting point for the elucidation of the molecular mechanisms and candidate genes for the functional analyses of hetero- and homo-graft systems in watermelon and generally in *Cucurbitaceae*.

## Data Availability Statement

The original contributions presented in the study are publicly available. This data can be found here: NCBI repository, accession number: PRJNA721571 (https://www.ncbi.nlm.nih.gov/bioproject/PRJNA721571).

## Author Contributions

FB, AP, AA, and AK: conceptualization, methodology, and data analysis. FB, GT, EM, and IT: experimental measurements. FB, GT, AP, and AK: writing—original draft preparation. AK: supervision and project administration. All authors: editing.

## Conflict of Interest

The authors declare that the research was conducted in the absence of any commercial or financial relationships that could be construed as a potential conflict of interest.

## Publisher’s Note

All claims expressed in this article are solely those of the authors and do not necessarily represent those of their affiliated organizations, or those of the publisher, the editors and the reviewers. Any product that may be evaluated in this article, or claim that may be made by its manufacturer, is not guaranteed or endorsed by the publisher.

## Footnotes

1
http://www.agrishorticulture.com

2
https://www.bioinformatics.babraham.ac.uk/projects/trim_galore/

3
http://daehwankimlab.github.io/hisat2/

4
https://ccb.jhu.edu/software/stringtie/

5
https://github.com/alyssafrazee/ballgown

6
https://github.com/trinityrnaseq/trinityrnaseq/wiki

7
https://busco.ezlab.org/

8
https://github.com/bbuchfink/diamond

9
https://www.ncbi.nlm.nih.gov/Traces/study/?acc=PRJNA721571

10
http://cucurbitgenomics.org/
